# Royal Jelly Ameliorates Behavioral Deficits, Cholinergic System Deficiency, and Autonomic Nervous Dysfunction in Ovariectomized Cholesterol-Fed Rabbits

**DOI:** 10.3390/molecules24061149

**Published:** 2019-03-22

**Authors:** Yongming Pan, Jianqin Xu, Ping Jin, Qinqin Yang, Keyan Zhu, Mengmeng You, Fuliang Hu, Minli Chen

**Affiliations:** 1 Comparative medical Research Institute, Experimental Animal Research Center, Zhejiang Chinese Medical University, Hangzhou 310053, China; pym@zcmu.edu.cn (Y.P.); xujianqing1@zcmu.edu.cn (J.X.); qqy1029@126.com (Q.Y.); 20151051@zcmu.edu.cn (K.Z.); 2 College of Animal Sciences, Zhejiang University, Yuhangtang Road 866, Hangzhou 310058, China; ymm0233@163.com; 3 The third clinical medical college, Zhejiang Chinese Medical University, Hangzhou 310053, China; jp830717@163.com

**Keywords:** royal jelly, cholinergic, autonomic nervous system, menopause, Alzheimer’s disease, bee products

## Abstract

Estrogen deficiency after menopause is associated with autonomic nervous changes, leading to memory impairment and increased susceptibility to Alzheimer’s disease (AD). Royal jelly (RJ) from honeybees (*Apis mellifera*) has estrogenic activity. Here, we investigated whether RJ can improve behavior, cholinergic and autonomic nervous function in ovariectomized (OVX) cholesterol-fed rabbits. OVX rabbits on high-cholesterol diet were administered with RJ for 12 weeks. The results showed that RJ could significantly improve the behavioral deficits of OVX cholesterol-fed rabbits and image structure of the brain. RJ reduced body weight, blood lipid, as well as the levels of amyloid-beta (Aβ), acetylcholinesterase (AchE), and malonaldehyde (MDA) in the brain. Moreover, RJ also increased the activities of choline acetyltransferase (ChAT) and superoxide dismutase (SOD) in the brain, and enhanced heart rate variability (HRV) and Baroreflex sensitivity (BRS) in OVX cholesterol-fed rabbits. Furthermore, RJ was also shown to reduce the content of Evans blue and the expression levels of Aβ, beta-site APP cleaving enzyme 1(BACE1), and receptor for advanced glycation end products (RAGE), and increase the expression level of LDL(low density lipoprotein) receptor-related protein 1 (LRP-1) in the brain. Our findings suggested that RJ has beneficial effects in neurological disorders of postmenopausal women, which were associated with reducing cholesterol and Aβ deposition, enhancing the estrogen levels and the activities of cholinergic and antioxidant systems, and ameliorating the blood–brain barrier (BBB) permeability and restoring autonomic nervous system.

## 1. Introduction

Decreased estrogen levels after menopause can lead to cognitive dysfunction and increasing the risk of Alzheimer’s disease (AD) [1]. In China, the incidence of AD in women is twice that in men [1], and vast majority of them are postmenopausal women. Animal studies also have shown that the loss of ovarian function was associated with altered brain metabolism and increased oxidative stress, and leads to accumulation of amyloid-beta (Aβ) [2]. In fact, gender-based differences may be the normality rather than the exception for the central nervous system (CNS) disorders [3]. The relative susceptibility of women to AD is not only associated with the loss of menopausal hormones, but also with the action of CNS. It was recently found that several central nervous structures mainly affected by AD were also involved in the function of autonomic nervous system (ANS), such as cerebral neocortex, insular cortex, brain stem, and hypothalamus, etc. [4,5]. In particular, the insular cortex is not only affected in the preclinical stage of AD, but also related to cardiac autonomic dysfunction [6]. Recent studies have demonstrated that the ANS function in premenopausal women is higher than in postmenopausal women, and estrogen can affect central and peripheral ANS by regulating sympathetic/parasympathetic nerves [7], suggesting that menopause is associated with ANS dysfunction.

It was confirmed by Allan et al. [8] that a disorder of ANS existed in dementia patients, and the underlying pathophysiological mechanism was caused by insufficient activity of the cholinergic system. The cholinergic system not only affects the parasympathetic nervous system and the sympathetic nervous system, but also all common dementias are associated with cholinergic deficits [9], which is mainly featured with a significant reduction of acetylcholine [10]. In addition, it has been found that estrogen can partially act through the cholinergic system to improve cognition [11]. Collins et al. [12] deemed that autonomic nervous dysfunction may exist before the clinical symptoms of AD. Therefore, recovering cholinergic system and autonomic nervous function may be a new therapeutic approach to treating the decline of memory behavior in postmenopausal patients, and thus prevent the occurrence of AD.

Although animal experiments and clinical studies have shown that estrogen improves postmenopausal cognitive function [13,14,15], the effectiveness of hormone replacement therapy (HRT) still remains controversial [16]. In particular, the Women’s Health Initiative Memory Study (WHIMS) found that the use of HRT in the late menopause failed to improve cognitive decline during neurodegenerative degeneration. Additionally, it increased the risk of breast cancer and cardiovascular disease [17]. Subsequent studies have shown that the reason for the ineffectiveness of WHIMS may be related to the treatment window of HRT, and it has been recommended to receive treatment in the early perimenopausal period or just after menopause [18,19].

Studies have verified that aging and decreased estrogen levels are related to autonomic nervous changes of postmenopausal women [20], whereas some studies have not shown that estrogen therapy has a significant effect on heart rate variability (HRV) of postmenopausal women [21,22]. HRV is a simple and non-invasive tool for describing fluctuations between continuous heartbeat intervals (RR intervals) that can be used to identify phenomena associated with the autonomic nervous system [23]. More importantly, the estrogen receptor (ER) possesses two different isoforms, ERα and ERβ. Studies have found that estradiol primarily improves memory deficits via activating ERβ instead of ERα [24,25]. On the other hand, activation of ERαis the main risk factor for cancer [26,27]. Hence, the risks of suffering from breast, endometrial, and ovarian cancer will be increased by HRT as a result [28,29]. Given the negative aspects of HRT, finding alternative and natural drugs will contribute to minimizing postmenopausal neurological disorders and improving cognitive behavioral disorders.

Royal jelly (RJ) from honeybees (*Apis mellifera)* has various pharmacological effects, including immunomodulation, anti-hypertensive, anti-fatigue, anti-tumor, and anti-osteoporosis [30,31,32].RJ contains fatty acids, lipids, proteins, carbohydrates, and vitamins. Several compounds from RJ have recently been found to have estrogenic activity. RJ contains fatty acids and sterols, including 10-hydroxy-trans-2-decenoic acid, 10-hydroxydecanoic acid, trans-2-decenoic acid, and 24-methylenecholesterol. These components have estrogenic activity and bind to ERβ more selectively rather than ERα [33,34]. Moreover, hypercholesterolemia is also associated with AD risk [35]. Not only have the cell culture results indicated that accumulation of cholesterol can lead to accelerated cleavage of amyloid precursor protein (APP) into amyloid protein [36], but animal experiments also showed that high cholesterol-fed rabbits can cause brain plaque formation and confirm Aβ deposition in plaque [37]. Jaya Prasanthi et al. [38] have confirmed that a high-cholesterol-diet-induced Aβ formation mechanism is associated with an increased expression of β-site APP cleaving enzyme 1(BACE1) and receptor for advanced glycation end products (RAGE) and a decreased expression of LRP1 in the rabbit brain. In this study, we investigated the protective effects of RJ on neurological disorders in ovariectomized (OVX) cholesterol-fed rabbits through behavior, blood biochemistry, autonomic nervous function, blood–brain barrier (BBB) permeability, magnetic resonance imaging (MRI), and pathological detection and analysis, providing experimental evidence for the application of RJ in the prevention of neurological disorders in menopause women.

## 2. Results

### 2.1. RJ Reduced Blood Lipid Levels in OVX Cholesterol-Fed Rabbit

We first observed the effects of RJ on body weight, uterine weight, and blood lipid levels in OVX cholesterol-fed rabbits. As shown in Table 1, the body weights in the high cholesterol diet (HCD) group and OVX + HCD group were significantly higher than that in the sham group after 12 weeks of modeling. Compared with OVX + HCD group, the body weight in OVX + HCD + RJ group was significantly reduced. The Estradiol (E_2_) and progesterone levels in the OVX + HCD group were significantly lower than those in the sham group and HCD group, whereas RJ intervention significantly increased the E_2_ and progesterone levels in OVX + HCD rabbits. Meanwhile, the levels of total cholesterol (TC), triglycerides (TG), low-density lipoprotein cholesterol (LDL-C), and high-density lipoprotein cholesterol (HDL-C) in the HCD group and OVX + HCD group were significantly higher than those in the sham group; whereas the levels of TC, TG, and LDL-C in the OVX + HCD + RJ group were significantly lower than those in the OVX + HCD group. In addition, there was no significant difference between the uterine weight in the HCD group and that in the sham group. However, the uterine weight in the OVX + HCD group was significantly lower than that in the sham group, and no marked difference in uterine weight between the OVX + HCD + RJ group and OVX + HCD group was observed.

### 2.2. RJ Improved Behavioral Deficits in OVX Cholesterol-Fed Rabbits

To assess the effects of RJ on behavioral deficits in OVX cholesterol-fed rabbits, the behavioral changes were detected by open field test in the four groups of rabbits. As shown in Table 2, the success rate of behavior of spontaneously searching food-water activity within 5 minutes and the response rate to sudden sound stimulation in the HCD and OVX + HCD groups were lower than those in the sham group, and those in the OVX + HCD group were decreased significantly. Nevertheless, compared with OVX + HCD group, the success rate of behavior of spontaneously searching food–water activity within 5 minutes and the response rate to sudden sound stimulation in the OVX + HCD + RJ group were significantly improved.

### 2.3. RJ Ameliorated BBB Permeability and Reduced Neuronal Loss in OVX Cholesterol-Fed Rabbits

**Figure 1:**
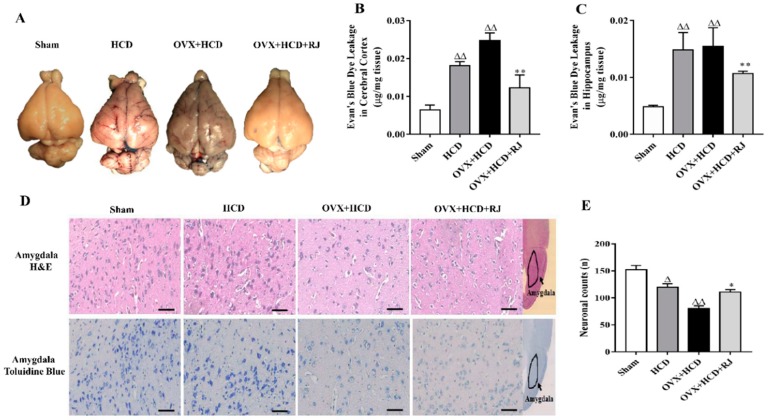
Royal jelly (RJ) ameliorated blood–brain barrier (BBB) and reduced neuronal loss in ovariectomized cholesterol-fed rabbits. (**A**) Representative photos of Evans blue staining in rabbit brains, and Evan’s blue dye leakage in the cerebral cortex (**B**) and hippocampus (**C**). (**D**) Representative photos of H&E staining and toluidine blue staining in the amygdala of rabbit brain, scale bar = 50 µm, and neuronal counts per view (40×) in the amygdala areas among the four groups (**E**), *n* = 4 rabbit per group. Data are presented as mean ± SEM. Compared with Sham group, ^Δ^*P* < 0.05, ^ΔΔ^*P* < 0.01; Compared with OVX + HCD group, * *P* < 0.05, ** *P* < 0.01.2.4. RJ enhanced heart rate variability (HRV) and baroreflex sensitivity (BRS) in OVX cholesterol-fed rabbits.

The BBB permeability was assessed using the Evans blue method in OVX cholesterol-fed rabbits. The results from Figure 1 showed that the Evans blue contents in the cerebral cortex and hippocampus of the HCD group and the OVX + HCD group were significantly higher than those of the sham group. Compared with the OVX + HCD group, the Evans blue content in the cortex and hippocampus of OVX + HCD + RJ group was significantly reduced. Moreover, the cell counts in the amygdala of HCD group and OVX + HCD group were markedly decreased with H&E staining and toluidine blue staining, nucleus pyknosis, and even vacuolization appeared in OVX + HCD group, while RJ significantly improved the morphological structure and number of neurons in OVX rabbits (*P* < 0.01, Figure 1D,E).

**Figure 2:**
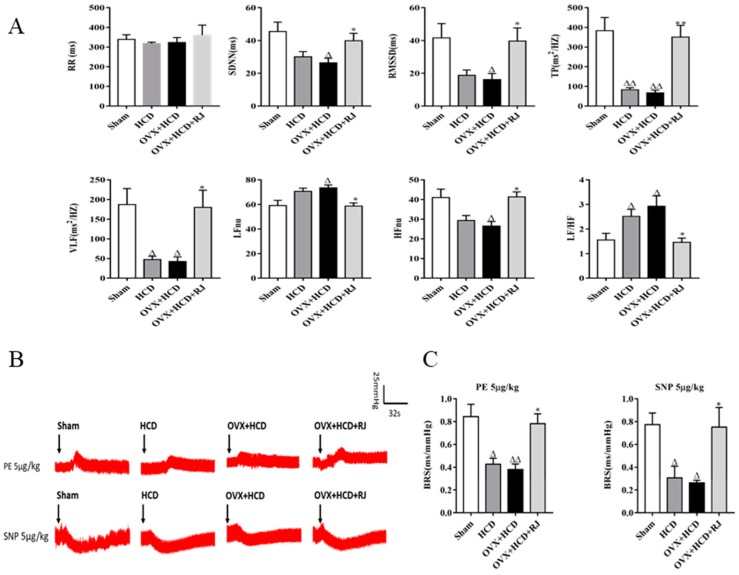
RJ enhanced HRV and BRS in ovariectomized cholesterol-fed rabbits. (**A**) The continuous heartbeat (RR) interval, standard deviation of NN (R-R) intervals (SDNN), the root mean square successive difference (RMSSD), total power (TP), very low-frequency power (VLF), normalized low-frequency (LF) nu, normalized high-frequency (HF) nu, and LF/HF ratio were analyzed in each group. The HRV parameters were significantly decreased in the HCD group and OVX + HCD group, while RJ treatment could significantly recover the balance of HRV. (**B**) Representative blood pressure response curve of each group induced by PE 5μg/kg and SNP 5μg/kg, and the changes of baroreflex sensitivity (BRS) value induced by PE or SNP (**C**) were observed in each group. The BRS values were significantly decreased in the HCD group and OVX + HCD group, while RJ treatment could significantly increase the BRS values. Data are expressed as mean ± SEM. *n* = 4–5. Compared with sham group, ^Δ^*P* < 0.05, ^ΔΔ^*P* < 0.01; Compared with OVX+HCD group, * *P* < 0.05, ** *P* < 0.01.

To assess the effects of RJ on ANS function in OVX cholesterol-fed rabbits, the heart rate variability (HRV) and baroreflex sensitivity (BRS) were detected in rabbits of the four groups. As shown in Figure 2A, compared with sham group, total power (TP) and very-low-frequency (VLF) were both significantly decreased in HCD group, while low-frequency (LF)/high-frequency (HF) ratio was significantly increased. Similarly, the standard deviation of the RR interval (SDNN), the mean square root of the adjacent RR interval (RMSSD), TP, VLF, and HFnu were significantly decreased in OVX + HCD group, and LFnu and LF/HF ratio were significantly increased. Conversely, RJ treatment significantly increased SDNN, RMSSD, TP, VLF, and HFnu and obviously reduced LFnu and LF/HF ratio in OVX cholesterol-fed rabbits. In addition, the phenylephrine (PE) or sodium nitroprusside (SNP)-induced BRS values in HCD group and OVX + HCD group were significantly lower than those in the sham group (Figure 2B, C). In contrast, RJ treatment could significantly increase PE or SNP-induced BRS values in OVX cholesterol-fed rabbits (Figure 2B,C).

### 2.4. RJ Regulated the Expression of LRP1/RAGE and Reduced BACE1 Activity and Aβ Deposition in the Brains of OVX Cholesterol-Fed Rabbits

**Figure 3:**
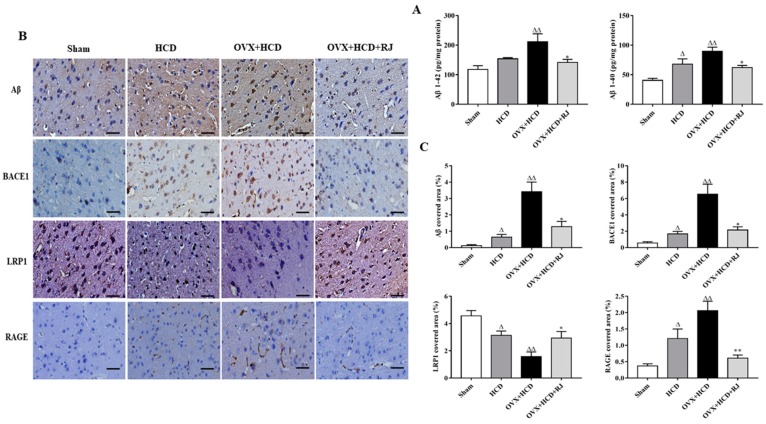
RJ regulated LRP1/RAGE expression and reduces BACE1 activity and Aβ deposition in ovariectomized cholesterol-fed rabbits. (**A**) The levels of Aβ1-40 and Aβ1-42 in the brain of each group measured by ELISA, *n* = 5 rabbits per group. (**B**) Staining for Aβ, BACE1, LRP1, and RAGE using specific antibody in sagittal sections of the brain. Scale bar = 50 µm. (**C**) The covered area of Aβ, BACE1, LRP1, and RAGE staining in the brain of each group. Data are expressed as mean ± SEM. *n* = 5. Compared with Sham group, ^Δ^*P* < 0.05, ^ΔΔ^*P* < 0.01; Compared with OVX + HCD group, * *P* < 0.05, ** *P* < 0.01.

To assess the effects of RJ on Aβ accumulation and its potential mechanism, the expression levels of Aβ, BACE1, LRP1, and RAGE were detected in the rabbit brains of each group by immunohistochemistry. Compared with the sham group, the Aβ 1-40 level in the brain of the HCD group was significantly increased, and the Aβ 1-40 and Aβ 1-42 levels in the brain of the OVX + HCD group were also significantly increased (Figure 3A). However, Aβ 1-40 and Aβ 1-42 levels in the RJ-treated group were significantly reduced in the brains of OVX cholesterol-fed rabbits (Figure 3A). In addition, compared with the sham group, the positive expression levels of Aβ, BACE1, and RAGE were significantly increased in the brains of the HCD group and the OVX + HCD group, while the positive expression level of LRP1 was significantly decreased (Figure 3B, C). The changes of the above indicators were significantly improved after RJ treatment. However, quantitative analysis displayed that RJ treatment significantly decreased the positive expression levels of Aβ, BACE1, and RAGE and increased the positive expression level of LRP1 in OVX cholesterol-fed rabbits (Figure 3B,C).

### 2.5. RJ Enhanced Cholinergic System Activities and Antioxidant Abilities in OVX Cholesterol-Fed Rabbit Brains

**Figure 4:**
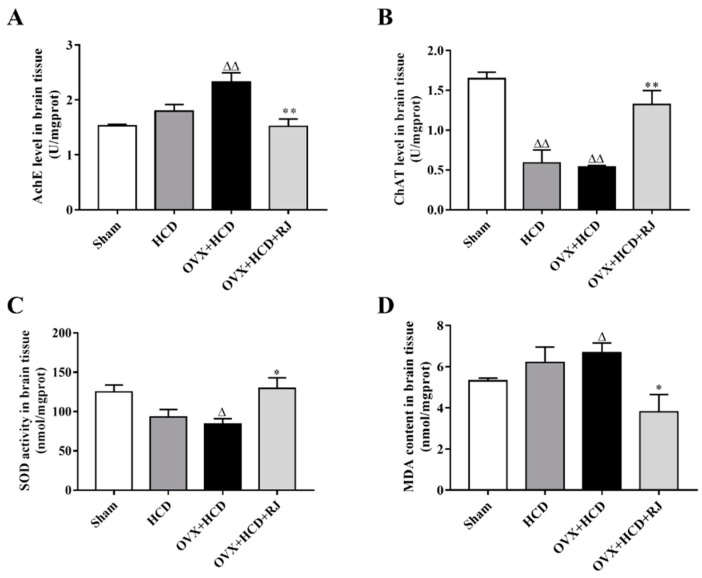
RJ enhanced cholinergic system activities and antioxidant abilities in the brain of ovariectomized cholesterol-fed rabbits. Ache level (**A**), ChAT level (**B**), SOD activity (**C**), and MDA content (**D**) were detected in the brain of each group. Data are expressed as mean ± SEM. *n* = 4–5. Compared with Sham group, ^Δ^*P* < 0.05, ^ΔΔ^*P* < 0.01; Compared with OVX + HCD group, * *P* < 0.05, ** *P* < 0.01.

The effects of RJ on cholinergic system activities and antioxidant abilities of OVX cholesterol-fed rabbit brains were measured. As shown in Figure 4, the choline acetyltransferase (ChAT) level was significantly reduced in the brain of the HCD group compared with the sham group. Similarly, the ChAT and superoxide dismutase (SOD) levels were markedly reduced in the brain of the OVX + HCD group compared with the sham group, while the acetylcholinesterase (AchE) activity and malonaldehyde (MDA) content were significantly increased in the brain of the OVX + HCD group. Moreover, RJ treatment could significantly reduce the AchE activity and MDA content, and increase the ChAT and SOD levels in OVX cholesterol-fed rabbits.

### 2.6. Royal Jelly Improved the Structure of OVX Cholesterol-Fed Rabbit Brains

**Figure 5:**
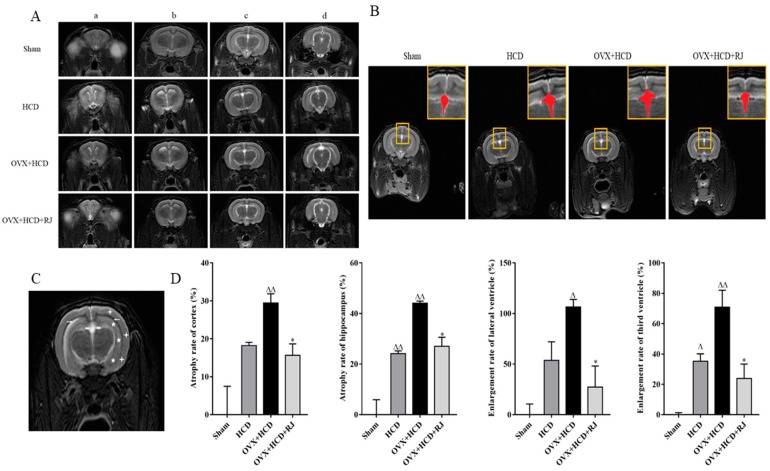
RJ improved the structure of brain MRI T2W image in ovariectomized cholesterol-fed rabbits. (**A**) T2W images on rabbit brain of OVX + HCD, HCD, OVX + HCD + RJ, and Sham group at different levels from left to right: the level of the rostral part of the hypophysis (**a**), the caudal part of the hypophysis (**b**), the thalamus (**c**), and the mesencephalic aqueduct (**d**).(**B**) The effect of RJ on the structure of the third ventricle in ovariectomized Alzheimer’s disease (AD) rabbits. Yellow square as enlarged view, red marks as the third ventricle area. (**C**) T2W image from a rabbit received high-cholesterol diet in ovariectomized rabbits. The cortex (white cross), hippocampus (white pentagram), the third ventricles (white dovetail arrow) and lateral ventricles (white arrow) were identified. (**D**) Quantitative analysis of changes in the cortex, hippocampus, lateral ventricle, and third ventricle in each group. The atrophy rate of cortex and hippocampus, as well as enlargement of the lateral ventricle and third ventricle, were detected by quantitative analysis. Data are expressed as mean ± SEM. *n* = 4. Compared with Sham group, ^Δ^*P* < 0.05, ^ΔΔ^*P* < 0.01; Compared with OVX + HCD group, * *P* < 0.05, ** *P* < 0.01.

We then used MRI to evaluate the effects of RJ on imaging structure of OVX cholesterol-fed rabbit brains. As shown in Figure 5A, the signal enhancement was observed in multiple areas at the junction of the hippocampus and the lateral ventricle regions in the HCD group and the OVX + HCD group, which was significantly reduced after RJ intervention. In addition, the third ventricles of the HCD group and the OVX + HCD group were obviously more dilated than that in the sham group, which was significantly reduced by RJ administration (Figure 5B). Moreover, quantitative analysis showed that the atrophy rate of hippocampus and the dilatation rate of third ventricular were significantly increased in the HCD group compared with the sham group (Figure 5C). Similarly, the atrophy rates of cortical and hippocampus and the dilatation rates of lateral ventricle and third ventricular were significantly increased (Figure 5C). In contrast, RJ treatment could significantly reduce the atrophy rates of the cortex and hippocampus and the dilatation rates of lateral ventricle and third ventricular in OVX cholesterol-fed rabbits (Figure 5C), indicating that RJ could significantly improve the imaging structure of OVX cholesterol-fed rabbit brains.

## 3. Discussion

**Figure 6:**
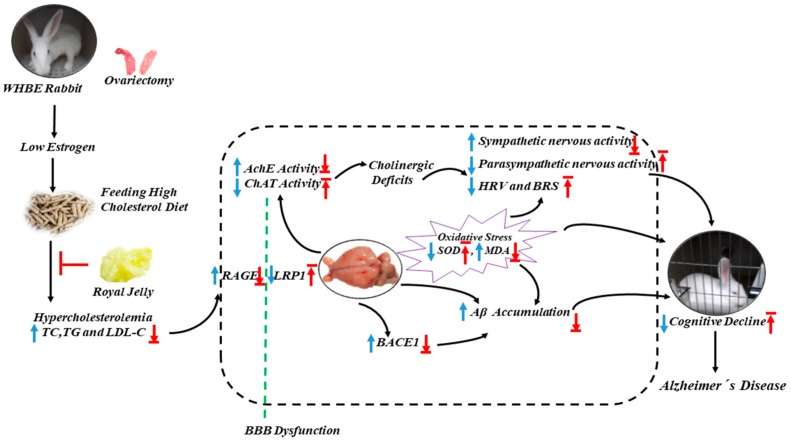
Schematic showing the possible mechanisms by which RJ improves OVX cholesterol-fed rabbits. RJ can reduce blood lipid levels, enhance cholinergic and antioxidant abilities, improve BBB and ANS, down-regulate BACE1 and RAGE expression, increase the expression levels of LRP1, promote the metabolism of Aβ, and thereby ameliorate behavioral deficits and reduce the susceptibility to AD. TC, total cholesterol; TG, triglycerides; LDL-C, low-density lipoprotein cholesterol; BACE1, β -site APP cleaving enzyme 1; BBB, blood–brain barrier; RAGE, receptor for advanced glycation end products; LRP1, LDL receptor-related protein 1; Aβ, amyloid β -protein; AchE, Acetylcholinesterase; ChAT, Choline Acetyltransferase; MDA, malondialdehyde; SOD, superoxide dismutase; HRV, heart rate variability; BRS, baroreflex sensitivity. Arrows pointing up or down indicate statistically significant increases or decreases (*P* < 0.05). Blue arrows indicate the changes in OVX cholesterol-fed rabbits, while red arrows indicate the treatment effects of RJ.

In this study, ovariectomy or high-fat-diets-induced animal models were shown to impair spatial memory behavior in the same way as other postmenopausal animal models. OVX cholesterol-fed rabbits exhibited sluggishness and cognitive dysfunction, amygdala neuron loss, obvious estrogen and progesterone decline, lipid metabolism disorder, oxidative stress aggravation and Aβ aggregation, cholinergic deficit, BBB permeability impairment, hippocampus and cortical atrophy, the third ventricle and lateral ventricle dilatations, as well as autonomic nervous dysfunction. Whereas estrogen-protected HCD rabbits showed slightly milder symptoms than OVX + HCD rabbits, confirming that low estrogen levels were associated with changes of postmenopausal autonomic nervous [20]. In contrast, RJ intervention can significantly lower blood lipid levels, reduce Aβ aggregation, restore estrogen levels and number of amygdala neurons, enhance cholinergic receptor activities and antioxidant capacities, and improve BBB permeability and ANS function, thereby improving brain structure and cognitive behavioral deficits (Figure 6).

The levels of estradiol and progesterone in OVX rabbits were significantly reduced but not completely depleted, which may be related with these sex hormones secreted in the adrenal cortex in addition to ovarian secretion. Anne Caufriez et al. [39] showed that the adrenal cortex is the only source of postmenopausal progesterone production, and the CYP19A1 enzyme in the adrenal cortex can catalyze the production of estradiol from testosterone [40]. The various components of RJ have estrogen-like effects, such as 10-hydroxy-2-olenoic acid, 10-hydroxy succinic acid, trans-2-ylenic acid, and 24-methylene cholesterol [41]. At the same time, it was also found that 10-HDA in royal jelly can promote the function of adrenal cortex [42]. In addition, previous studies have found that RJ intervention can increase steroid hormones and estrogen levels [43] and improve plasma progesterone levels in sheep [44]. Therefore, we considered that RJ intervention can significantly increase the levels of estradiol and progesterone in OVX rabbits, which may be related to the secretion of endogenous sex hormones in the adrenal cortex by RJ. More importantly, supplementation of estradiol and progesterone has been shown to improve mild cognitive impairment in menopausal women [45], and this may be the mechanism by which RJ is beneficial for improving neurological disorders. However, the activation of ERα is a major risk factor for cancer [26,27]. In ERα-knockout mice, E2 does not increase uterine weight [46]. Our results also showed that RJ had no significant affect the reduction of body weight and increment of uterine weight in OVX cholesterol-fed rabbits, which was consistent with Suzuki’s report [33]. These results indicated that RJ hardly had any effect on ERα, which may be a beneficial aspect of RJ.

Previous studies have shown that oral administration of 6 g or 10 g RJ per day can significantly lower cholesterol levels or increase HDL-C Level in human clinical treatment [47,48], and animal experimental studies also have shown that daily administration of 700 mg/kg RJ can reduce the cholesterol level in rats [49]. Furthermore, Chiu et al. [50] concluded that the major RJ protein (MRJP) component in RJ can reduce blood lipids in patients with mild hypercholesterolemia by improving the dehydroepiandrosterone sulphate (DHEA-S) levels. According to the clinical and animal dose conversion relationship, our study also showed that the administration of 400 mg/kg RJ to OVX rabbits also significantly reduced cholesterol levels, confirming that RJ has a lipid-lowering effect. In addition, high cholesterol level is a factor of increasing Aβ generation and Aβ toxicity [51,52]. Previous studies have also shown an increase in brain Aβ deposition in postmenopausal women or ovariectomized mice [13,53], which was consistent with our observations in OVX + HCD rabbits. Moreover, elevated Aβ levels caused by the high cholesterol diet was closely related to up-regulation of BACE1 expression [38]. Conversely, estrogen can down-regulate the BACE1 activity through the MARK /ERK signaling pathway, reduce the production of Aβ, and promote the degrading of Aβ by activating the phagocytosis and degradation of microglia [54]. In this study, the expression level of BACE1 was significantly up-regulated in the brains of OVX + HCD rabbits, which may be related to dyslipidemia induced by low estrogen level after the menopause [55]. It was also found that the levels of Aβ and BACE1 in the brain of Sham and HCD rabbits with estrogen protection were lower than those of OVX + HCD rabbits, and yet we found that RJ could significantly reduce the body weight, blood lipid, Aβ level as well as the expression of BACE1, thereby inhibiting the production of Aβ in OVX cholesterol-fed rabbit brains, suggesting that estrogen supplementation can inhibit the production of Aβ in early postmenopausal women.

The amygdala plays a key role in emotional anxiety. Kawasaki K et al.’s [56] studies have shown that the number of neurons in the basolateral amygdala of anxiety rats is significantly reduced, which is consistent with our results observed in the OVX rabbits, and RJ can reverse these pathological changes. Moreover, since RJ contains fatty acids and sterols with estrogenic activity acting on ERβ [33,34], the beneficial effects of RJ on behavioral deficits may be caused via ERβ activation. The low-polarity fatty acids and sterols in RJ may be favorable for RJ to cross the BBB and play a beneficial role in the brain. Estrogen not only lowers the cholesterol levels and protects Aβ neurotoxicity, but also regulates Aβ metabolism. Aβ can cross the BBB in both directions via LRP and RAGE, which may play a key role in regulating brain Aβ and plaque formation [57]. Furthermore, studies have shown that rabbits with high cholesterol diet have impaired BBB and enhanced permeability, making plasma cholesterol more likely to enter the CNS, increasing brain cholesterol level and inducing increased Aβ production [58]. In this study, abnormal BBB function was observed in the cholesterol-fed intact rabbits and OVX rabbits. However, the degree of BBB dysfunction of HCD rabbits with estrogen protection is less severe than that of OVX rabbits. Furthermore, we also found that RJ could reduce the Evans blue content, increase the expression level of LRP1 and inhibit the expression level of RAGE in the cholesterol-fed OVX rabbit brain, which may be beneficial to reduce cholesterol metabolism disorder, improve the permeability of BBB, and regulate the translocation of Aβ, thus reducing Aβ deposition in the brain.

Cognitive function is associated with the cardiac autonomic nervous function, and cognitive performance is negatively correlated with sympathetic function and positively correlated with parasympathetic function [59]. Significant parasympathetic dysfunction exists even in individuals with mild cognitive impairment [12]. The parasympathetic nervous system releases acetylcholine through the vagus nerve to affect heart rate, resulting in bradycardia. This reduction of parasympathetic nervous system activity is often identified as vagal withdrawal, indicating that the autonomic nervous function was impaired [60]. HRV and BRS are two commonly used methods for assessing autonomic nervous function. HRV is significantly reduced among menopausal women [61]. In addition, BRS is a crucial mechanism for maintaining human circulatory stability. Clinical studies have shown that cardiovascular BRS disorders are associated with poor memory [62]. Impaired HRV and BRS may lead to blood pressure dysregulation or orthostatic hypotension, resulting in cerebral hypoperfusion and cognitive decline [63]. In turn, neurodegenerative processes could affect cardiac autonomic dysfunction [64,65]. In the present study, compared with high cholesterol-fed intact rabbits, OVX cholesterol-fed rabbits exhibited more excessive sympathetic state (such as increased LFnu and LF/HF ratio) and lower vagal state (such as decreased RMSSD and HFnu) relatively, and BRS was significantly decreased, leading to serious cognitive behavioral deficits, which are consistent with autonomic dysfunction in menopausal women [61,66]. Furthermore, we found that RJ could enhance HRV and BRS, thereby improving cognitive behavioral deficits.

The possible mechanism by which RJ restores autonomic nervous function is related to the enhancement of the cholinergic system. Some evidence demonstrated that the autonomic dysfunction is due to cholinergic deficiency [67], and cardiac autonomic function changes may be a symbol of central nervous system dysfunction. Changes of the levels of cholinergic neurotransmitters in the central nervous system are significantly associated with learning and memory ability. ACh is an important neurotransmitter that promotes both memory and cognition. Ach is first synthesized by ChAT and decomposed by AchE to maintain the dynamic balance of Ach in the brain. Once the ChAT or AchE activity is abnormal, it will lead to learning and memory decline [68]. Studies have shown a significant increase in hippocampal AChE activity in ovariectomized animals [69]. Additionally, oxidative stress is involved in the process of cognitive function [70]. Reducing or increasing endogenous antioxidant enzyme activity may contribute to memory loss or enhancement [71], and estrogen has an anti-oxidative effect [72], which is consistent with no obvious changes in oxidative stress parameters in HCD rabbits with estrogen protection. In addition, this study also confirmed that ChAT and SOD activities were significantly decreased, and AchE and MDA contents were significantly increased in OVX cholesterol-fed rabbit brains, indicating the presence of cholinergic deficiency and oxidative stress. However, RJ can significantly enhance the cholinergic system and antioxidant capacities, which is beneficial to improve the autonomic nervous system and cognitive behavioral disorders.

## 4. Materials and Methods

### 4.1. Laboratory Animals

A total of 24 female White Hair and Black Eyes (WHBE) rabbits (4–5 months old, 2.0–2.4 kg) were purchased from Xin Jian rabbit field (Certificate No. SCXK, Zhejiang, 2015-0004, China). They were singly caged under a controlled 12-h light/dark cycle and had free access to feed and water. The animals were cared for in accordance with the guidelines established by the Laboratory Animal Research Center of Zhejiang Chinese Medical University (Certificate No. SYXK, Zhejiang, 2013-0184). All animal experiments were pre-approved by the animal ethics committee of the Zhejiang Chinese Medical University (IACUC Approval No. ZSLL-2016-115).

**Figure 7:**
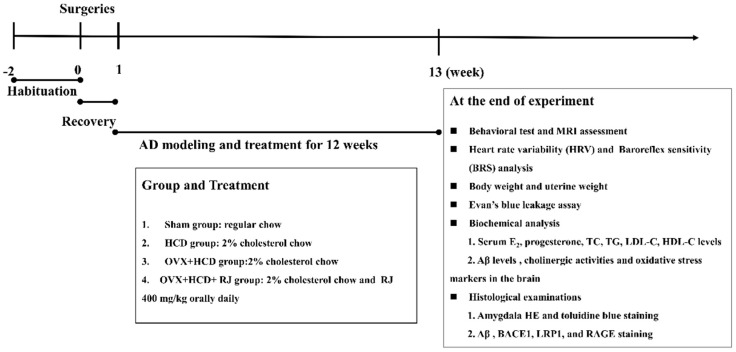
A flowchart of the experimental design in this study.

After adaptation to the environment for 2 weeks, all rabbits were randomly divided into 4 groups (6 rabbits in each group): sham group, high cholesterol diet (HCD) group, ovariectomy (OVX) + HCD group, and OVX + HCD + RJ group. Rabbits were subjected to either sham or ovariectomized surgery, and they were fed normal chow diet plus 2% (*w/w*) cholesterol (120 grams per day) on the 7th day after surgery lasting for 12 weeks, except for the sham group (fed with the normal chow). Meanwhile, the RJ-treated group received 400 mg/kg RJ (obtained from Jiangshan, Zhejiang, China) via oral administration every day for 12 weeks. This selected dose of RJ refers to the previous clinical data that an oral administration of 6 g RJ per day can significantly reduce cholesterol levels in human clinical therapy [47]. After the rabbits were sacrificed, the uteri were resected and weighed. The experimental designs are shown in Figure 7. After 12 weeks of administration, the behavioral test, MRI assessment, a set of biochemical index detection in serum and brain, HRV measurement, BRS test, Evan’s blue leakage assay, as well as pathological indices observation were performed.

### 4.2. Blood Lipid Levels Measurement

After 12 weeks of modeling, all rabbits fasted for 12 h, and then 2 mL blood samples were drawn from their middle auricular artery with heparin anticoagulation, from which blood plasma was separated. The changes of TC, TG, LDL-C, and HDL-C levels in blood samples were analyzed using automatic biochemical analyzer (7020, HITACHI, Japan). The specific procedures were carried out, following the kit instructions in accordance with each indicator (Shanghai Shenneng-DiaSys Diagnostic Technology Co., Ltd., China). The levels of E_2_ and progesterone in plasma were quantified by using an ELISA kit (Jiancheng Bioengineering Institute, Nanjing, China).

### 4.3. Behavioral Test

After all rabbits in each group fasted for 24 h, the rabbit behavioral test was carried out using the wild plain method according to Xu’s report [73]. Briefly, a quiet and open environment with natural light was selected to conduct the test. The field used was an activity area with the rectangular square of 60 cm × 60 cm as a unit, the grids of 3 × 5, and the area of 5.4 m^2^, surrounded by baffles. During the examination, chow and drinking water were fixed in a place of the field, and the number of rabbits who discovered the food-drinking water within 5 minutes in each group was recorded. Each rabbit was subjected to a fixed audio noise stimulation during the test, and the behavioral reaction of the rabbit responding to noise stimuli was observed.

### 4.4. Evans Blue Permeability Determination 

Evans blue was used as a tracer to measure the BBB permeability [74]. Firstly, 2% Evans blue (2 mL/kg) had been injected intravenously into the ear vein an hour before killed, and then the whole bodies of animals were observed to turn blue rapidly, which was continued to circulate for 60 min. Next, rabbits were anesthetized with 3% sodium pentobarbital solution (30mg/kg), and the chest was immediately opened to expose the heart. Then, the left ventricle of the heart was carefully perfused with pre-cooled normal saline and right atrium was opened as well. Finally, the brain was taken promptly after colorless perfusion was observed, of which the left and right cerebral cortex and hippocampus were removed prudently. Meanwhile, each brain tissue above was divided into two parts accordingly, weighted, and stored in formamide for 72 h at room temperature in the dark. The samples were then centrifuged at 10,000× *g* for 10 min, and the supernatants were collected to measure OD values at 620 nm. At the same time, OD values of Evan’s blue solution were measured at gradient concentrations of 2.5, 5, 10, 50, and 100 μg/mL, and the standard curve was drawn. Accordingly, the content of Evans blue was calculated, expressed as Evan’s blue concentration/sample weight (μg/mg tissue).

### 4.5. Electrocardiogram (ECG) Detection and Heart Rate Variability (HRV) Analysis 

At 12 weeks of modeling, the chest hair of rabbits in each group was shaved. Subsequently, the naked chest was attached with ECG pads and connected to ECG electrodes. After rabbits were put on telemetry jackets, their Lead II ECG signals in free and awake states were continuously monitored using EMKA non-invasive physiological signal telemetry system (EMKA, France) for 2 h. Then, HRV time-domain analysis was performed, and the indicators included the RR interval, SDNN, and RMSSD. In addition, frequency domain power spectrum analysis was performed according to the method previously studied [75], setting the VLF power as 0–0.0625 Hz, the LF power as 0.0625–0.1875 Hz and the HF power as 0.1875–2 Hz. Analysis indexes included TP, VLF, LF, and HF. Afterward, LF, HF (LFnu or HFnu = LF or HF / (TP-VLF)) and LF/HF ratios were standardly calculated. HF is believed to represent the rapid activity of the parasympathetic nervous system, whereas the LF is believed to reflect both sympathetic and parasympathetic activities. Moreover, LF/HF reflects the index of sympathovagal balance.

### 4.6. Baroreflex Sensitivity (BRS) Analysis

At the end of the experiment, each rabbit was anesthetized by inhalation of 2–3% isoflurane–oxygen mixture delivered via the mask, and anesthesia was maintained by using 0.5–2% isoflurane–oxygen mixture. During the anesthesia, the median neck incision was performed to separate the right common carotid artery. Then, the catheter fully filled with 50 U/mL heparin sodium was inserted into the artery and connected to a pressure transducer. After that, changes of blood pressure curve were recorded by MedLab-U/8C biosignal acquisition system (Nanjing Meiyi Company, China). BRS was detected by intravenous injection of phenylephrine (PE) or sodium nitroprusside (SNP) into ear border vein of the rabbit to raise or lower blood pressure, causing changes in the reflective RR interval. BRS value is represented by the ratio of changing the value of RR interval and changing the value of systolic blood pressure (SBP). According to the pre-experimental results, PE of 5 μg/kg was selected to inject intravenously, which would raise blood pressure and cause reflex bradycardia, reflecting the sensitivity of pressure reflection to elevated blood pressure. When the blood pressure returned to the basal level after injection, SNP 5 μg/kg was injected via the ear vein to decrease blood pressure and reflexively cause tachycardia, reflecting the sensitivity of pressure reflection to reduced blood pressure.

### 4.7. Determination of Aβ Level by ELISA

The levels of Aβ1-40 and Aβ1-42 in the rabbit cortex and hippocampus were measured following the kit instructions. Brain tissue supernatants were prepared referring to previous research methods [38] and then quantified using ELISA kit (Jiancheng Bioengineering Institute, Nanjing, China). The protein concentration of all samples was determined via adopting BCA assay method (Pierce, Rockford, IL, USA) [76]. Aβ levels were normalized to the total protein content of the samples.

### 4.8. Determination of AchE, ChAT, SOD, and MDA Contents in Brains

The activities of AchE, ChAT, and SOD and contents of MDA in the brain were measured according to the procedures of colorimetric commercial kit (Jiancheng Bioengineering Institute, Nanjing, China). The SOD activity was examined with xanthine oxidase method, and the MDA content was examined with sulfur barbituric acid method [77]. Briefly, part of the brain tissue was taken, to which 4 °C cold saline solutions were added proportionally. Next, 5% *w/v* brain homogenate was obtained using a homogenizer (IKA-werke Gmbh Co., KG, German), which was centrifuged at 2095× *g* for 20 min at 4 °C. Then, the supernatant was aspirated and stored at −80 °C for use. The protein concentration was determined by Coomassie brilliant blue method, and all indexes above were normalized by the total protein content of the samples.

### 4.9. Histopathological Examination

All rabbits were euthanized with 3% pentobarbital sodium (30 mg/kg), and then their hearts were perfused with 300 mL 4 °C PBS solutions. After cardiac perfusion, the brain tissue was removed and then fixed in formalin solution for at least 24 h. The selected slices were dehydrated with gradient ethanol, embedded in paraffin, and segmented into sections with 6 μm thickness. The sections were subjected to H&E staining and 1% toluidine blue staining to observe the cell morphology and number of neurons in the amygdala. In addition, the expression of Aβ, BACE1, LRP1, and RAGE in brain tissue was observed by immunohistochemistry. Paraffin sections were first roasted in a 60 °C incubator for 1 h, then dewaxed to water, and finally microwave heat repair was performed as follows. In brief, repair liquid was poured into a beaker, which was heated in the microwave to boil twice, 1 min every time. After cooled with running water, sections were blocked with 3% H_2_O_2_ solution for 10 min, rinsed with PBS for 3 min, and then incubated with primary antibodies overnight at 4 °C, including β-amyloid (B-4,1:100, Santa Cruz Biotechnology, USA, which recognizes APP and Aβ), BACE1 (1:100, Santa Cruz Biotechnology, USA), LRP1 (1:300, Diage Biological Technology co. Ltd., China), and RAGE (1:100, Santa Cruz Biotechnology, USA). PBS was used as the negative control instead of the primary antibody. After that, the sections were washed with PBS three times, incubated with secondary antibody for 1 h at room temperature, visualized with DAB (ZSJQ, Beijing, China), and counterstained with hematoxylin. Brownish yellow or light yellow represented the positive substance. Three fields of view in each section were photographed continuously at 10 × magnification, and the staining percentage of the positive area in the whole visual cortex was measured using Image-pro plus 6.0 software.

### 4.10. MRI Scanning

MRI examination was performed at 12 weeks of modeling, and signals were received using a 3.0T MRI scanner (GE Discovery MR 750, GE, USA) coupled with an 8-channel rabbit specialized coil (Shanghai Chenguang Medical Technology Co., Ltd., China). Rabbits were anesthetized by intramuscular injection of 30 mg/kg ketamine and 4 mg/kg xylazine 15 min before imaging. After the anesthesia was stabilized, rabbits were fixed in the prone position. During the examination, the body temperature was maintained at 36–37 °C, and breathing was kept smooth. The parameters of coronal T2W Image scan sequence were as follows: TR = 5500 ms, TE = 100 ms, slice thickness = 3 mm, field of view (FOV) = 10 mm × 10 mm, matrix = 352 × 256, NEX = 2. Changes of brain imaging structure of all rabbits in T2W Image were observed. The areas of cortex, hippocampus, lateral ventricle, and third ventricle in brains were measured by IPP 6.0 software. The sham group was used as the standard to calculate the enlargement or atrophy of the brain structure of each group.

### 4.11. Statistical Analysis

All data were expressed as mean ± SEM. Statistical analyses were performed using one-way ANOVA with Tukey’s multiple comparisons test or chi-Square test was for counting data with Graphpad Prism 6.0 (GraphPad Software Inc.). *P* < 0.05 indicated statistical significance.

## 5. Conclusions

The results of the present study provide experimental evidence that RJ may have beneficial effects in neurological disorders of postmenopausal, which can ameliorate behavioral deficits via reducing blood lipid levels, enhancing estrogen levels, cholinergic activities, and antioxidant abilities, improving BBB and ANS, and promoting the metabolism of Aβ. This study also shows that estrogen therapy can improve postmenopausal cognitive dysfunction and may reduce the susceptibility to AD after menopause. Therefore, we expect to further study of the effect of RJ on AD.

## Figures and Tables

**Table 1:** Changes of body weight, uterine weight, and lipid metabolism in each group at 12 weeks.

Parameters	Sham	HCD	OVX + HCD	OVX + HCD + RJ
Body Weight (kg)	2.70 ± 0.06	3.03 ± 0.07^ΔΔ^	3.05 ± 0.04^ΔΔ^	2.84 ± 0.03*
Uterine Weight (g)	11.63 ± 0.31	11.75 ± 0.36	7.46 ± 0.62^ΔΔ^	7.27 ± 0.43
E_2_ (ng/L)	65.04 ± 4.91	63.86 ± 12.61	25.39 ± 8.71^ΔΔ^	50.79 ± 6.36*
Progesterone (ng/mL)	2.90 ± 0.47	2.50 ± 0.29	1.06 ± 0.41^Δ^	2.15 ± 0.19*
TC (mmol/L)	1.73 ± 0.22	48.57 ± 4.61^ΔΔ^	51.55 ± 11.83^ΔΔ^	30.00 ± 2.94*
HDL-C (mmol/L)	0.49 ± 0.04	2.98 ± 0.12^ΔΔ^	2.86 ± 0.09^ΔΔ^	2.47 ± 0.10
LDL-C (mmol/L)	1.02 ± 0.19	35.23 ± 2.94^ΔΔ^	35.29 ± 4.24^ΔΔ^	22.76 ± 2.59*
TG (mmol/L)	0.61 ± 0.07	1.24 ± 0.18^Δ^	2.07 ± 0.77^ΔΔ^	1.12 ± 0.16*

HCD: high cholesterol diet; OVX: ovariectomized; E2: estradiol; TC: total cholesterol; HDL-C: high-density lipoprotein cholesterol; LDL-C: low-density lipoprotein cholesterol; TG: triglycerides. Data are presented as mean ± SEM from 6 rabbits in each group. Compared with sham group, ^Δ^*P* < 0.05, ^ΔΔ^*P* < 0.01; Compared with OVX + HCD group, * *P* < 0.05, ** *P* < 0.01.

**Table 2:** Changes in searching food–water and sound stimulus responses in each group.

Group	N	Spontaneously Searching Food–Water Behavior Within 5 Minutes			Sudden Sound Stimulus Response		
		Success	Failure	Successful Rate (%)	Yes	No	Response Rate (%)
Sham	6	6	0	100	6	0	100
HCD	6	3	3	50%	3	3	50%
OVX + HCD	6	0	6	0%^ΔΔ^	0	6	0%^ΔΔ^
OVX + HCD + RJ	6	5	1	83.3%*	5	1	83.3%*

Chi-square test, compared with sham group, ^Δ^*P* < 0.05, ^ΔΔ^*P* < 0.01; Compared with OVX+HCD group, * *P* < 0.05, ** *P* < 0.01.

## Abbreviations

AD	Alzheimer’s disease
Aβ	Amyloid-beta
CNS	Central nervous system
HRT	Hormone replacement therapy
ER	Estrogen receptor
RJ	Royal jelly
APP	Amyloid precursor protein
MRI	Magnetic resonance imaging
HCD	High cholesterol diet
OVX	Ovariectomy
E2	Estradiol
TC	Total cholesterol
TG	Triglycerides
LDL-C	Low-density lipoprotein cholesterol
HDL-C	High-density lipoprotein cholesterol
BBB	Blood-brain barrier
ECG	Electrocardiogram
HRV	Heart rate variability
SDNN	Standard deviation of the RR interval
RMSSD	The mean square root of the adjacent RR interval
VLF	Very-low-frequency
LF	low-frequency
HF	high-frequency
BRS	Baroreflex sensitivity
PE	Phenylephrine
SNP	Sodium nitroprusside
SBP	Systolic blood pressure
AchE	Acetylcholinesterase
ChAT	Choline Acetyltransferase
SOD	Superoxide dismutase
MDA	Malonaldehyde
BACE1	beta-site APP cleaving enzyme 1
LRP1	LDL receptor-related protein 1
RAGE	Receptor for advanced glycation end products
